# A new approach methodology to identify tumorigenic chemicals using short-term exposures and transcript profiling

**DOI:** 10.3389/ftox.2024.1422325

**Published:** 2024-10-17

**Authors:** Victoria Ledbetter, Scott Auerbach, Logan J. Everett, Beena Vallanat, Anna Lowit, Gregory Akerman, William Gwinn, Leah C. Wehmas, Michael F. Hughes, Michael Devito, J. Christopher Corton

**Affiliations:** ^1^ Center for Computational Toxicology and Exposure, US Environmental Protection Agency, Durham, NC, United States; ^2^ Oak Ridge Associated Universities (ORAU), Oak Ridge, TN, United States; ^3^ National Institute of Environmental Health Sciences (NIEHS), Division of Translational Toxicology, Durham, NC, United States; ^4^ U.S. Environmental Protection Agency, Office of Pesticide Programs, Washington, DC, United States

**Keywords:** new approach methodologies, biomarkers, liver cancer, transcript profiling, adverse outcome pathway, 2-year cancer bioassay

## Abstract

Current methods for cancer risk assessment are resource-intensive and not feasible for most of the thousands of untested chemicals. In earlier studies, we developed a new approach methodology (NAM) to identify liver tumorigens using gene expression biomarkers and associated tumorigenic activation levels (TALs) after short-term exposures in rats. The biomarkers are used to predict the six most common rodent liver cancer molecular initiating events. In the present study, we wished to confirm that our approach could be used to identify liver tumorigens at only one time point/dose and if the approach could be applied to (targeted) RNA-Seq analyses. Male rats were exposed for 4 days by daily gavage to 15 chemicals at doses with known chronic outcomes and liver transcript profiles were generated using Affymetrix arrays. Our approach had 75% or 85% predictive accuracy using TALs derived from the TG-GATES or DrugMatrix studies, respectively. In a dataset generated from the livers of male rats exposed to 16 chemicals at up to 10 doses for 5 days, we found that our NAM coupled with targeted RNA-Seq (TempO-Seq) could be used to identify tumorigenic chemicals with predictive accuracies of up to 91%. Overall, these results demonstrate that our NAM can be applied to both microarray and (targeted) RNA-Seq data generated from short-term rat exposures to identify chemicals, their doses, and mode of action that would induce liver tumors, one of the most common endpoints in rodent bioassays.

## 1 Introduction

In the United States, cancer is the second leading cause of death, imposing a tremendous burden on individuals and their families, as well as the US economy (Ahmad and Anderson, 2021; CDC, 2017). Most chemicals in commerce have not been adequately tested for the ability to cause cancer in humans and animals. The 2-year cancer bioassay conducted in mice and rats remains the “gold standard” for carcinogenicity testing, but due to the resources needed to assess a chemical ($2–4 M USD; 800 rodents; histopathological analysis of more than 40 tissues; 2+ years to complete the in-life study and years to analyze results), only ∼1500 commercial chemicals have been examined to date (Bucher and Portier, 2004; Gold et al., 2005; Waters et al., 2010). In contrast, there are tens of thousands of chemicals in commerce with inadequate information on cancer hazard. These include over 140,000 substances registered by the European Registration, Evaluation, Authorization and Restriction of Chemicals (REACH) (REACH, 2008), ∼30,000 chemicals being used commercially in the United States and Canada (Muir and Howard, 2006), and ∼41,000 chemicals on the US EPA’s Toxic Substances Control Act Inventory (https://www.epa.gov/tsca-inventory; accessed 1 August 2022). There are also concerns about the human relevance of rodent cancer outcomes. New resource-efficient methods are needed to move away from reliance on the 2-year cancer bioassay and to identify the carcinogenic potential of a chemical in shorter term *in vivo* assays or through sets of assays carried out in appropriate *in vitro* systems allowing identification of human-relevant risk that can be put into the context of boundaries of exposure.

There are increased efforts across broad sectors of the toxicity testing community to develop new approach methodologies (NAMs) to reduce or entirely replace animal testing. The Organization of Economic and Cooperative Development (OECD) (Jacobs et al., 2020), institutions in the United States (ICCVAM, 2018; Sciences, 2018) (ICCVAM 2018; NIEHS 2018; Hood 2019; U.S. EPA 2020a), and the European Union (Annys et al., 2014; Corvi et al., 2017; Luijten et al., 2020) have efforts to replace the rodent chronic bioassay using more human-relevant testing methods, that if implemented will significantly reduce or replace animal testing (Felter et al., 2021). The NAMs being developed and validated can include relevant *in vitro* assays that do not use animals as well as *in vivo* studies that are for shorter durations of exposure and use fewer animals per treatment group than the 2-year bioassay (Cohen et al., 2019; Madia et al., 2019). Some NAMs can already be used to help predict human carcinogenic risk in a regulatory setting including *in silico* mutagenicity prediction models used to classify an impurity of concern in an active pharmaceutical ingredient and reduce further testing to assess carcinogenic risk (ICH (2017) M7 regulations). Additionally, activities are ongoing to include weight-of-evidence for the carcinogenicity assessments for agrochemicals (Hilton et al., 2022) and pharmaceuticals (Bourcier et al., 2024) which incorporate all available relevant data. The past work highlights the considerable challenges to using NAMs to accurately predict human cancer risk including what endpoints to measure *in vitro* assays and when to measure them. Although NAMs are starting to be used and/or considered by some regulatory agencies (Jacobs et al., 2020; Luijten et al., 2020; Yauk et al., 2020; Heusinkveld et al., 2020), there is currently limited regulatory acceptance for decision-making.

Genomic biomarkers are being increasingly recognized by broad sectors of the scientific community to have the potential to reduce the need for conventional rodent carcinogenicity studies of chemicals through a weight-of-evidence approach. Biomarker-based NAMs could be used in integrated approaches to testing and assessment (IATA) strategies or could be used as standalone NAMs for an intended use (Corton et al., 2022a). Gene expression biomarkers have been developed and applied for hazard identification in a number of contexts. One of the first biomarkers to be developed was the TGx-DDI biomarker, currently under regulatory review by the FDA through the Center for Drug Evaluation and Research Biomarker Qualification Program (Avila et al., 2020). The biomarker was developed to enable differentiating between true positive DNA damage-inducing (DDI) agents and non-DDI irrelevant positive agents using a number of human cell lines (Li et al., 2017; Corton et al., 2018; Cho et al., 2019). Another set of biomarkers were developed to identify molecular initiating events (MIEs) in cancer and liver steatosis adverse outcome pathways (AOPs) by leveraging microarray data from livers of chemically-treated wild-type and transcription factor-null mice, allowing for the identification of well-defined mechanistic gene sets (Oshida et al., 2015a; Oshida et al., 2015b; Oshida et al., 2016; Rooney et al., 2018a; Rooney et al., 2018b; Rooney et al. 2,019). These biomarkers have been applied to sets of chemicals to identify relationships between exposure and hazard (Rosen et al., 2017), as well as to identify the most likely AOP responsible for rodent liver tumors (Peffer et al., 2018; Rooney et al., 2017; Rooney et al., 2018c). Given the growing emphasis on tiered screening of chemicals using high-throughput transcriptomics (HTTr) in human cell lines (Thomas et al., 2019; Harrill et al., 2021), a number of groups have constructed biomarkers that identify important molecular targets underpinning *in vivo* toxicity including estrogen receptor (ER) (Ryan et al., 2016) and androgen receptor (Rooney J. P. et al., 2018) modulation as well as stress factor induction (Jackson et al., 2020; Rooney et al., 2020; Cervantes and Corton, 2021; Korunes et al., 2022) and histone deacetylase inhibition (Cho et al., 2021; Corton et al., 2022b). Future NAMs may one day use gene expression biomarkers used to interpret transcript profiles derived from *in vitro* HTTr human-derived multicellular models (micro-physiological systems, organoids, organ-on-a-chip) that better mimic the physiological and toxicological behaviors of human organs compared to the current screening paradigm carried out in two dimensional human cell cultures. Like NAMs, regulatory acceptance of biomarker use for toxicological assessments is rare; only the GARDskin/GARDpotency used to identify skin sensitizers in a human myeloid dendritic-like cell line have been accepted for regulatory studies (OECD TGP 4.106).

In the present study, we describe and test the predictive capability of a NAM using a set of gene expression biomarkers, that was designed to meet 21st century goals of reduced reliance on animals to identify potential carcinogens using short-term exposures in rats and transcript profiling. The NAM was trained to not only identify chemicals and their doses that would cause rat liver tumors but to also identify the underlying chemical mode of action (MOA). Regulatory agencies would then be able to use prior knowledge to determine if the MOA is of human relevance and whether the chemical would need to be examined in a 2-year bioassay. As the NAM was trained and tested on microarray data, we determined if the NAM would be able to accurately identify liver tumorigens using (targeted) RNA-Seq data. We found that the NAM accurately identifies chemicals and their doses that cause liver tumors in rat chronic studies under a wide variety of acute testing conditions. The information derived from the NAM then can be used to determine if the predicted mode of action would be relevant to humans.

## 2 Methods

### 2.1 Overview of datasets used in the analysis

There were three datasets used in our analysis which are described in greater detail below.

• “Study A”: To confirm that our approach could be used to identify hepatotumorigens using Affymetrix data at a single dose and time, we utilized a dataset generated in male Sprague-Dawley rats exposed to 22 chemicals at a single dose level each day for 4 days (rat 4-day study). This study has not been previously described. (Quick summary: 22 chemicals; 1 dose; 4 days; Affymetrix)
• “Study B”: To compare biomarker activation levels generated using Affymetrix and RNA-Seq, we utilized a published dataset that was generated in male Sprague-Dawley rats exposed to 27 chemicals at one dose level each day for 3, 5 or 7 days (rat Affymetrix-RNA-Seq comparison study) derived from the DrugMatrix study. The livers of the rats were evaluated for gene expression changes using Affymetrix arrays and in later studies by RNA-Seq. The data from this study came from (Bushel et al., 2018; Svoboda et al., 2019; Wang et al., 2014). (Quick summary: 27 chemicals; 1 dose; 3, 5, or 7 days; comparing Affymetrix vs. RNA-Seq)
• “Study C”: To determine if the biomarker tumorigenic activation levels (TALs) generated using microarray data could be applied to targeted RNA-Seq data, we used a dataset generated in male Sprague-Dawley rats exposed to 16 chemicals at up to 10 dose levels for 5 days (rat 5-day study). The livers of the rats were evaluated for gene expression changes using TempO-Seq. The livers used in this study came from a previously published study (Gwinn et al., 2020). (Quick summary: 16 chemicals; up to 10 doses; 5 days; targeted RNA-seq). It should be noted that the 5-day study was available to us to use as a dataset to determine if the Tempo-Seq platform could be used in the NAM, not to optimize the minimal number of doses to be used.

### 2.2 Rat 4-day study (study A)

#### 2.2.1 Chemicals

The chemicals and doses used in the study are found in Table 1 and include those that are carcinogenic and noncarcinogenic at the doses used. There were also a set of chemicals in which the carcinogenic status of the dose used is not known. The following chemicals were obtained from Bayville Chemical Supply Corporation (Deer Park, NY) at label purities >95%: acetochlor, ametryn, cyclanilide, cyfluthrin, cyprodinil, flusilazole, indoxacarb, simazine, and tebufenpyrad. The following chemicals were obtained from Sigma-Aldrich (St Louis, MO) at label purities >97%: bisphenol A, carbaryl, ethyl methanesulfonate, flutamide, lipopolysaccharide, perfluorooctanoic acid, and triclosan. WY-14,643 was obtained from A.G. Scientific (San Diego, CA) at a label purity of 98.5%, and estragol was obtained from Penta Manufacturing Company (Livingston, NJ) at a label purity of 99.7%. Lipopolysaccharide was obtained from Sigma-Aldrich Company (St. Louis, MO). Syringeability, solubility, and concentration were verified for each chemical using either HPLC or GC methodologies.

**TABLE 1:** Chemicals used in the rat 4-day study (study A).

Common chemical name (abbreviation used in the study)	CASRN	DTXSID	Dose level used in the study (mg/kg/day)	Dose classification^1^	Lowest tumorigenic dose (mg/kg/day)	Highest non-tumorigenic dose (mg/kg/day)
2,5-Pyridinedicarboxylic acid, dipropyl ester	136-45-8	DTXSID8032544	600	3	1000	500
Acetochlor	34256-82-1	DTXSID8023848	250	1	250	69
Ametryn	834-12-8	DTXSID1023869	176	1	176	26.2
Bisphenol A	80-05-7	DTXSID7020182	450	3		95.4
Carbaryl	63-25-2	DTXSID9020247	100	2	500	100
Cyclanilide	113136-77-9	DTXSID5032600	58.6	3		43.1
Cyfluthrin	68359-37-5	DTXSID5035957	23	2		23
Cyprodinil	121552-61-2	DTXSID1032359	74	2		73.6
Di (2-ethylhexyl) phthalate	117-81-7	DTXSID5020607	600	1	99	19.8
Estragole	140-67-0	DTXSID0020575	600	1	600	
Ethyl methanesulfonate	62-50-0	DTXSID6025309	200	3		
Flusilazole	85509-19-9	DTXSID3024235	13	3		
Flutamide	13311-84-7	DTXSID7032004	10	3		50
Indoxacarb	173584-44-6	DTXSID1032690	10	2		10
Lipopolysaccharride (LPS)	NOCAS_36695	DTXSID4036695	2	3		
N,N-dimethyl-p-toluidine	99-97-8	DTXSID0021832	60	1	60	20
Perfluorooctanoic Acid	335-67-1	DTXSID8031865	15	1	4	1.9
Pirixinic acid (WY-14,643)	50892-23-4	DTXSID4020290	10	1	10	
Simazine	122-34-9	DTXSID4021268	63	2		1000
Tebufenpyrad	119168-77-3	DTXSID0034223	17	1	6.5	
Triclosan	3380-34-5	DTXSID5032498	1000	3		127
Vinclozolin	50471-44-8	DTXSID4022361	225	1	225	83

^1^Tumorigenicity classification of the dose used in the study: 1 = tumorigenic; 2 = not tumorigenic; 3 = not known.

#### 2.2.2 Rat exposures

Male Harlan Sprague-Dawley rats (Harlan Laboratories, Dublin, VA) (6–9 weeks old) were maintained on a 12-h light/dark cycle at 20-25°C with a relative humidity of 30%–70%, fed NTP-2000 diet (Zeigler Bros., Gardners, PA) and provided food and water *ad libitum*. Rats were housed individually during acclimation and grouped 2 per cage. Animals were assigned to a dose group using a procedure that stratifies animals across groups by body weight such that mean body weight per group did not differ statistically among groups at the start of the study based on analysis of variance (ANOVA) (Statistical Analysis System version 9.1, SAS Institute, Cary, NC). Each vehicle control and treatment group had 6 animals. Studies were run on blocks of 4–6 chemicals at a time, with a common group of vehicle-treated animals for comparison.

Rats were exposed daily to either a 1% acetone/99% corn oil vehicle or test chemical (Table 1) dissolved in vehicle for four consecutive days by oral gavage at a dosing volume of 5 mL/kg. Ethyl methanesulfonate was administered in saline. Lipopolysaccharide was dissolved in saline and administered only once by intraperitoneal injection, 4 h prior to terminal sacrifice. For these last two chemicals, saline was used as the control. Dose volumes were adjusted for body weight daily. The dose of test chemicals used was in most cases based on the liver tumorigenic doses from cancer bioassays. For chemicals that did not cause rat liver tumors, the highest dose in the bioassay was used. To convert the dietary exposures to daily oral gavage exposures, average daily dietary intake was estimated from individual studies based on food intake and chemical concentrations in the diet. This dose was then converted to an oral gavage dose. The dose of flutamide used was based on pilot studies examining its anti-androgenic effects in rats (data not shown).

At 1 and 4 h post dosing, animals were observed cage side. Four hours (±15 min) after the final dose administration, animals were humanely euthanized by CO_2_ asphyxiation and blood was collected via cardiac puncture. Death was confirmed by exsanguination. Rats were euthanized in the same order as they were dosed. Livers were excised and weighed. The left lobe of the liver was cut into cubes, flash frozen in liquid nitrogen, placed in cryotubes on dry ice, and then stored at or below −70°C for transcriptomic analysis. All animal procedures were in compliance with the Animal Welfare Act Regulations, 9 CFR 1–4. All animals were handled and treated according to the Guide for the Care and Use of Laboratory Animals (Clark et al., 1997).

#### 2.2.3 RNA isolation

Frozen liver samples (approximately 20–30 mg) were submerged in ten volumes of pre-chilled RNAlater®-ICE (Life Technologies, Carlsbad CA) and stored at −20°C ± 10°C for a minimum of 16 h. The RNAlater®-ICE supernatant was then removed and each liver tissue sample, weighing between 23.6 and 30.0 mg, was added to lysis buffer and homogenized using plastic disposable pestles (Fisher Scientific, Pittsburgh, PA). Following homogenization, samples were stored at −80°C ± 10°C until RNA was isolated. Samples were thawed and centrifuged. RNA was extracted from the supernatant, subjected to DNase I digestion, and isolated using the Qiagen RNeasy Mini Kit (Qiagen, Valencia, CA). Each RNA sample was analyzed for quantity and purity by UV analysis using a NanoDrop ID-1000 Spectrophotometer (NanoDrop Technologies, Wilmington, DE). Purity was defined as the ratio of A_260_ to A_280_; an acceptable purity range was defined as a value between 1.80 and 2.20. A minimum concentration of 35 ng/μL was targeted to ensure reliable amplification using Affymetrix GeneChip^®^ reagents and kits. All samples yielded an acceptable purity and concentration appropriate for use with the Affymetrix GeneChip^®^ 3′ IVT Express Kit. All samples were evaluated for RNA integrity using an RNA 6000 Nano Chip kit with an Agilent 2100 Bioanalyzer (Agilent, Santa Clara, CA) and were based on the RNA integrity number (RIN) calculated by the 2100 Expert software. A RIN value of 8 and above was met for all samples indicating ideal integrity for microarray processing.

#### 2.2.4 Microarray analysis

Total RNA (100 ng), isolated from each of the rat liver samples, was used to synthesize single-stranded DNA, which was subsequently converted into a double-stranded cDNA template for transcription. An *in vitro* transcription (IVT) reaction, which incorporates biotinylated ribonucleotide analogs, was then used to create labeled amplified RNA (aRNA). This RNA target preparation was performed using the Affymetrix GeneChip^®^ 3′ IVT Express Kit (Affymetrix Inc., Santa Clara, CA). All incubation steps during this preparation were completed using an Eppendorf Mastercycler^®^ thermal cycler (Eppendorf Hamburg, Germany).

Labeled aRNA was fragmented and subsequently hybridized to the Affymetrix Rat Genome 230 2.0 Array (31,099 probe sets) using an Affymetrix GeneChip^®^ Hybridization Oven 645. Washing and staining of the arrays was completed using the Affymetrix GeneChip^®^ Hybridization Wash and Stain kit and performed using the Fluidics Station 450 according to the Affymetrix recommended protocol. After washing and staining, the arrays were scanned using an Affymetrix GeneChip^®^ Scanner 3000 7G and the raw microarray data (.cel files) were acquired using Affymetrix GeneChip^®^ Command Console^®^ Software (AGCC). The following QC parameters were evaluated for each array: average background, scale factor, percent of genes scored as present, 3′ to 5′ ratios for the internal control genes beta-actin and glyceraldehyde-3-phosphate dehydrogenase (*Gapdh*), values for hybridization control transcripts, and values for poly (A) controls. Microarrays were normalized in GeneSpring 12.0 using RMA and features were then filtered in which a feature needed to be present at >20% percentile rank in the normalized intensity data in all samples from at least one treatment group. Filtered gene lists were then subject to a Welch test (unpaired, unequal variance *t*-test; treated vs. paired vehicle control). Genes with statistically significant differential expression were those exhibiting a *p*-value <0.05. The *p*-values were not subjected to a multiple testing correction, because this is not a standard applied for creating lists of differentially expressed genes in BaseSpace Correlation Engine (BSCE) (Kupershmidt et al., 2010). The genes exhibiting significant differential expression were further filtered by removing genes that exhibited less than an absolute 1.2-fold change. Lists of differentially expressed genes and their fold change values for each chemical treatment were uploaded into BSCE.

### 2.3 Rat affymetrix-RNA-Seq comparison study (study B)

The analysis of the profiles generated from this study have been described previously (Bushel et al., 2018; Svoboda et al., 2019; Wang et al., 2014). Briefly, male Sprague-Dawley rats were exposed by oral gavage to one of 27 chemicals at one dose level for 3, 5 or 7 days (three rats per chemical with matched controls). Liver RNA was isolated and analyzed using Affymetrix microarrays (Gene Expression Omnibus (GEO) accession number: GSE47875) and Illumina RNA-Seq (GSE55347). The chemicals and their doses used in the study are found in Table 2. Starting with the raw expression data available in GEO, all statistically filtered gene sets from the study were generated using the BSCE analysis pipeline for Affymetrix or RNA-Seq data that has been described previously (Kupershmidt et al., 2010).

**TABLE 2:** Chemicals used in (study B).

Chemical name	CASRN	DTXSID	Dose (mg/kg/day)
3-Methylcholanthrene	56-49-5	DTXSID0020862	300
Aflatoxin B1	1162-65-8	DTXSID9020035	0.3
17beta-Estradiol	50-28-2	DTXSID0020573	150
5,6-Benzoflavone	6051-87-2	DTXSID8030423	1500
Bezafibrate	41859-67-0	DTXSID3029869	617
Carbon tetrachloride	56-23-5	DTXSID8020250	1175
Cerivastatin	145599-86-6	DTXSID9022786	7
Chloroform	67-66-3	DTXSID1020306	600
Clofibric acid	882-09-7	DTXSID1040661	448
Clotrimazole	23593-75-1	DTXSID7029871	89
Econazole	27220-47-9	DTXSID2029872	334
17alpha-Ethinylestradiol	57-63-6	DTXSID5020576	10
Fluconazole	86386-73-4	DTXSID3020627	394
Gemfibrozil	25812-30-0	DTXSID0020652	700
Ifosfamide	3778-73-2	DTXSID7020760	143
Leflunomide	75706-12-6	DTXSID9023201	60
Lovastatin	75330-75-5	DTXSID5020784	450
Methimazole	60-56-0	DTXSID4020820	100
Miconazole	22916-47-8	DTXSID6023319	920
Nafenopin	3771-19-5	DTXSID8020911	338
N-Nitrosodimethylamine	62-75-9	DTXSID7021029	10
Norethindrone	68-22-4	DTXSID9023380	375
Phenobarbital	50-06-6	DTXSID5021122	54
WY-14,643	50892-23-4	DTXSID4020290	364
Rosiglitazone	122320-73-4	DTXSID7037131	1800
Simvastatin	79902-63-9	DTXSID0023581	1200
Thioacetamide	62-55-5	DTXSID9021340	200

### 2.4 Rat 5-day study (study C)

This study has been described previously (Gwinn et al., 2020). Briefly, male Sprague Dawley (Hsd: Sprague Dawley SD) rats were exposed by oral gavage to 16 chemicals at up to 10 doses once per day for 5 consecutive days (Days 0–4) with *n* = 4 rats per exposure concentration and vehicle control. The rats were sacrificed on the 5th day. The chemicals and their doses used in the study are found in Table 3. In the original study, the liver RNAs were evaluated using the rat S1500^+^ TempO-Seq platform. To comprehensively evaluate transcriptional benchmark dose (BMD) approaches, the RNAs used in the original study were re-isolated and evaluated using the rat full genome TempO-Seq platform. For RNA isolation, frozen RNA stabilized tissues were obtained from the National Toxicology Program, thawed on ice and ∼10 mg liver were distributed at one sample per well in nuclease-free 96-well plates (Cat. 89218-298, VWR, Radnor, PA, United States) preloaded with 50 µL/well RNAlater™ Stabilization Solution (Cat. AM7021, Invitrogen by ThermoFisher Scientific, Vilnius, Lithuania). Plates were sealed with nuclease-free aluminum seal (Cat. 75805-268, VWR^®^ Aluminium Foil Seals, Radnor, PA, United States) suitable for ultracold storage and stored at < −70°C until RNA isolation and purification was performed (BioSpyder Technologies, Carlsbad, CA, United States). For RNA isolation and purification, samples were processed using the RNAdvance purification kit (Beckman Coulter, Indianapolis, IN, United States) according to the manufacturer protocol. First, tissues were removed from RNAlater™ and transferred to deep-well homogenization plates loaded with RNAdvance lysis buffer and two stainless steel balls. Following homogenization, sample supernatants were digested in lysis buffer and RNA bound to kit provided SPRI beads. Bound RNAs underwent several rounds of incubation and washing followed by DNAse treatment according to RNAdvance protocol with purified RNA eluted in 40 µL nuclease-free water. Purified RNA was stored at < −70°C until sequenced. Raw TempO-Seq reads were aligned to all known probe sequences for the Rat Whole Transcriptome v1.0 probe set, as described previously (Harrill et al., 2021) (Everett et al., 2024 in preparation). Individual samples with <50% of reads uniquely aligned to known probe sequences, or <1 million uniquely aligned reads were removed from further analysis. Outlier samples were identified using PCA plots for each chemical and removed from further analysis as previously described (Everett et al., 2024 in preparation). For each chemical, differential expression analysis was performed using DESeq2 as previously described (Harrill et al., 2021). Briefly, probe counts were tabulated for all samples passing the quality checks described above, corresponding to each dose group and study-matched vehicle controls. Only those probes with mean count ≥5 were used for DESeq2 analysis. A single DESeq2 model was fit per chemical, with each dose group considered as an additional treatment factor. *p*-values and fold-changes were then computed for each dose group *versus* the vehicle control group. To derive a gene list for each dose group, genes were filtered to those with unadjusted *p*-value ≤0.05 (Wald test), and normal shrinkage was used to derive moderated log2 fold-change values. The gene lists were imported into BSCE and compared to the 6 biomarkers as described below. Outlier samples were removed as described (https://www.epa.gov/etap). There was one outlier removed in the following groups: DE71, 15 mg/kg; TBBPA, 31.25 mg/kg.

**TABLE 3:** Chemicals used in (Study C).

Chemical	Abbreviation	CASRN	DTXSID#	Dose levels (mg/kg/day)	Highest nontumorigenic dose	Lowest tumorigenic dose	Dose classification (in order of dosing order)^1^
Acrylamide	ACR	79-06-1	DTXSID5020027	0.075, 0.156, 0.3125, 0.625, 1.25, 2.5, 5, 10	2.7		2,2,2,2,2,3,3,3
Bromodichloroacetic acid	BDCA	71133-14-7	DTXSID4024644	1.25, 2.5, 5, 10, 20, 40, 80, 160	43		2,2,2,2,2,3,3,3
Coumarin	COU	91-64-5	DTXSID7020348	3.125, 6.25, 12.5, 25, 50, 100, 200, 400	71.4	200	2,2,2,2,2,3,1,1
Di (2-ethylhexyl) phthalate	DEHP	117-81-7	DTXSID5020607	8, 16, 31.25, 62.5, 125, 250, 500, 1000	19.8	99	2,2,3,3,1,1,1,1
Pentabromodiphenyl ether mixture	DE71	32534-81-9	DTXSID2024246	0.38, 0.75, 1.5, 3, 15, 50, 100, 200, 500	15	50	2,2,2,2,1,1,1,1
Ethinyl estradiol	EE2	57-63-6	DTXSID5020576	0.02, 0.067, 0.2, 0.6, 1.8, 5.4, 16.2, 48.6		0.429	3,3,3,1,1,1,1,1
Fenofibrate	FEN	49562-28-9	DTXSID2029874	8, 16, 31.25, 62.5, 125, 250, 500, 1000	10	45	2,3,3,1,1,1,1,1
Furan	FUR	110-00-9	DTXSID6020646	0.125, 0.25, 0.5, 1, 2, 4, 8, 16		1.4	2,2,2,2,2,3,1,1
Hexachlorobenzene	HCB	118-74-1	DTXSID2020682	0.004, 0.015, 0.0625, 0.25, 1, 4, 16, 64	1.6	5	2,2,2,2,2,3,1,1
Methyl eugenol	MET	93-15-2	DTXSID5025607	4.625, 9.25, 18.5, 37, 75, 150, 300, 600		26.4	3,3,3,1,1,1,1,1
Perfluorooctanoic acid	PFOA	335-67-1	DTXSID8031865	0.156, 0.3125, 0.625, 1.25, 2.5, 5, 10, 20		2.2	3,3,3,3,1,1,1,1
Pulegone	PUL	89-82-7	DTXSID2025975	2.4, 4.7, 9.4, 18.75, 37.5, 75, 150, 300	37.5		2,2,2,2,2,3,3,3
Tetrabromobisphenol A	TBBPA	79-94-7	DTXSID1026081	4, 8, 16, 31.25, 62.5, 125, 250, 500, 1000, 2000	1000		2,2,2,2,2,2,2,2,2,3
3,3′,4,4′-Tetrachloroazobenzene	TCAB	14047-09-7	DTXSID6026086	0.1, 0.3, 1, 3, 10, 30, 100, 200, 400	10	100	2,2,2,2,2,3,1,1,1
Tris (chloropropyl) phosphate	TCPP	13674-84-5	DTXSID5026259	18.75, 37.5, 75, 150, 300, 600, 1000, 2000	395	789	2,2,2,2,2,3,1,1
α,β-Thujone	THU	76231-76-0	DTXSID3040774	1.5, 3, 6.25, 12.5, 25, 50, 100, 200	50		2,2,2,2,2,2,3,3

^1^Tumorigenicity classification of the dose used in the study: 1 = tumorigenic; 2 = not tumorigenic; 3 = not known.

### 2.5 Determination of hepatocarcinogenicity of chemicals

We utilized a number of databases that had annotations for tumor induction after chronic exposure in rats. Most of the data came from the Lhasa Carcinogenicity Potency Database (CPD) (https://carcdb.lhasalimited.org/). Data for pesticides not in the CPD came from annotations in the ToxRef database (Watford et al., 2019) or National Toxicology Program studies. Carcinogenicity information for fenofibrate was kindly provided by Drs. Frank Sistare and Rachel Hao using the Pharmapendium database (https://www.elsevier.com/solutions/pharmapendium-clinical-data; accessed 13 August 2022). For all chemicals, we annotated effects described in these studies after chronic exposure on incidence of the following liver effects: hepatocellular carcinomas and adenomas, multiple liver tumor types, neoplastic nodules, trabecular hepatocellular carcinomas, and hepatocellular cholangiocarcinomas. The dose ranges and associated incidences were used to determine the highest non-tumorigenic dose and the lowest tumorigenic dose (if relevant). Any incidences greater than 5% over the control were considered tumorigenic, especially if higher doses resulted in greater incidences. Chemicals evaluated using the 2-year bioassay in which there were no increases in liver tumor incidences were assigned a highest non-tumorigenic dose representing the highest dose used in the study. For the most part, data was collected from 2-year bioassays. For one chemical (WY-14,643), only 1-year studies were available but allowed the derivation of lowest tumorigenic dose levels. Annotations were only made for chemicals with clear positive or negative responses, in female or male rats from any strain. All tumor data used in the analysis is found in Supplementary Files S1, S2.

### 2.6 Comparison of established biomarkers to gene lists

The six biomarkers for AhR, CAR, PPARα, ER, cytotoxicity and genotoxicity have been previously described (Rooney et al., 2018a; Hill et al., 2020). The biomarker genes and associated fold-changes along with the gene lists generated from the 4-day and 5-day rat studies described above were uploaded into BaseSpace Correlation Engine (BSCE), in which internal protocols rank the genes by absolute fold-change (Kupershmidt et al., 2010). The Running Fisher test is then used to compare the ranked biomarker genes to each ranked gene list from the three studies, calculating a pair-wise correlation *p*-value for the genes that overlap between lists. The *p*-values were converted to -Log (*p*-values) and negative correlations were converted to negative numbers. These procedures allowed the evaluation of the correlation of the overlaps between gene lists. Thus, the higher the -Log (*p*-value), the greater the correlation.

### 2.7 Application of tumorigenic biomarker activation levels

The activation levels of each of the biomarkers associated with tumorigenicity were derived as described earlier (Corton J. et al., 2020). Briefly, biomarker activation levels associated with liver tumor induction were derived from two large datasets: the TG-GATES study and the DrugMatrix study. Because we are at an early stage in potential use of the NAM, we wished to determine if one set of tumorigenic activation levels (TALs) are more predictive than another. Using chemical-dose pairs annotated for liver tumorigenicity, biomarker activation levels associated with the maximum -Log (*p*-value)s that did not generate a liver tumorigenic response were used as the TALs. The levels were derived from CodeLink microarray data from the DrugMatrix study or Affymetrix data from the TG-GATES study. The biomarker TALs are found in Supplementary File S3. Each biomarker -Log (*p*-value) derived from the three studies described above was evaluated relative to the biomarker TG-GATES and DrugMatrix TALs resulting in 12 tumorigenic biomarker activation levels to determine if exposure to a dose of a chemical exceeded or not the biomarker activation level. The datasets used to determine the TALs were not used in the present study. If any biomarker in each set of six exceeded the TAL, then the dose was predicted to lead to liver tumors in chronic studies, otherwise the dose was not predicted to be tumorigenic.

### 2.8 Determination of accuracy of the approach

For Study A and Study C, the predictive accuracy was determined at the level of the individual chemical-doses. For Study C, predictions were also made by chemical at any dose level. Predictions in this scenario would be similar to those used in preliminary testing of a new chemical entity to assist in avoiding any potential liabilities. The biomarker TALs derived from the TG-GATES or DrugMatrix studies (described above) were used to determine if the test chemical-dose exceeded or not the activation levels. The predictions for tumorigenicity were assigned a score of false positive (FP), false negative (FN), true positive (TP), or true negative (TN). Regarding the predictions based on any dose of a chemical (Study C), FN was assigned if all of the doses for a tumorigenic chemical were beneath all biomarker TALs. FP was assigned if any dose for a nontumorigenic chemical exceeded one or more of the biomarker TALs. These were the equations used in determining scores: balanced or predictive accuracy = (sensitivity + specificity)/2; sensitivity (TP rate) = TP/(TP + FN); specificity (TN rate) = TN/(FP + TN); positive predictive value (PPV) = TP/(TP + FP); negative predictive value (NPV) = TN/(TN + FN).

## 3 Results

### 3.1 Use of a NAM computational model to identify liver tumorigens after short-term exposures

Our study was designed to achieve three objectives. First, we wished to confirm that our NAM approach could be used to identify liver tumorigens when examining only one dose level. In this case, the study was conducted using Affymetrix arrays. The second objective was to compare the transcriptional responses between Affymetrix and RNA-Seq to determine if the derived biomarker -Log (*p*-value)s would be different between the two methods that may preclude accurate predictions using (targeted) RNA-Seq. Lastly, we wished to determine if the NAM could be applied to transcript profiles derived from (targeted) RNA-Seq (TempO-Seq) analyses without having to rederive the TALs.

**FIGURE 1:**
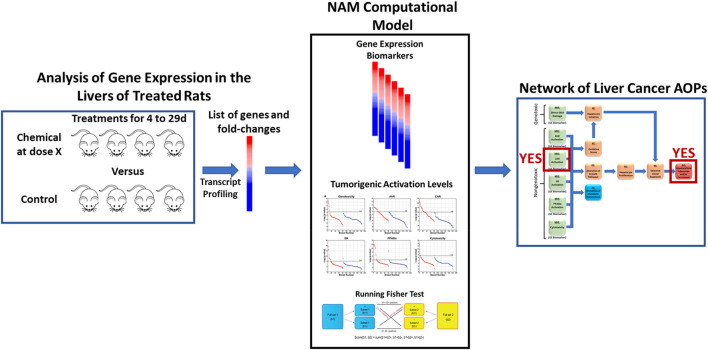
Use of a new approach methodology computational model to identify liver tumorigens. The computational model consists of three major components. There are six well-characterized gene expression biomarkers predictive of the modulation of the major molecular initiating events of rodent liver cancer. Each biomarker consists of 7–113 genes and associated fold-change values. The model includes biomarker activation levels associated with liver tumor induction after chronic exposure. The Running Fisher test within the BaseSpace Correlation Engine environment is used to compare each of the biomarkers to a transcript profile derived from the liver of a rat exposed to a new chemical entity for up to 29d. The model is able to identify MIEs activated and whether the level of activation exceeds a tumorigenic level.

To accomplish these objectives, we utilized a previously described NAM that can predict liver cancer outcomes using transcript profiles derived from the livers of rats treated with chemicals with unknown potential to cause liver cancer (Figure 1). The computational model consists of three major components necessary for prediction. First, there are six well-characterized gene expression biomarkers predictive of the modulation of the major MIEs of rodent liver cancer. Each biomarker consists of 7–113 genes and associated fold-change values that are used to determine whether a chemical is an activator of one or more MIEs (Rooney et al., 2018b; Corton J. C. et al., 2020; Corton J. et al., 2020; Hill et al., 2020; Lewis et al., 2020). As it is well known that activation by itself is not sufficient to generate the signals that lead to the adverse outcome, we had previously identified activation levels for each biomarker associated with tumor induction (called tumorigenic activation levels or TALs). We derived the TALs to predict induction of hepatocellular adenomas and/or carcinomas. The TALs have not been tested for other types of liver tumors, e.g., cholangiocarcinomas in part due to their rarity as outcomes. The last component is the Running Fisher statistical test within the BaseSpace Correlation Engine environment used to compare each of the biomarkers to the chemical-induced transcript profiles. We had previously determined that gene lists derived from the livers of rats exposed to a chemical up to 29d could be used by the NAM for accurate prediction or tumorigenicity (Rooney et al., 2018c; Corton J. C. et al., 2020; Corton J. et al., 2020; Hill et al., 2020; Lewis et al., 2020). Our previous studies showed that the predictions coming from the NAM computational model can not only be used to identify which MIEs are activated but whether the level of activation exceeds a tumorigenic level. Here, we apply this NAM to rat studies that vary by chemical, dose level, time of exposure, and profiling platform.

### 3.2 Prediction of tumorigenicity of chemicals examined at a single dose

**FIGURE 2:**
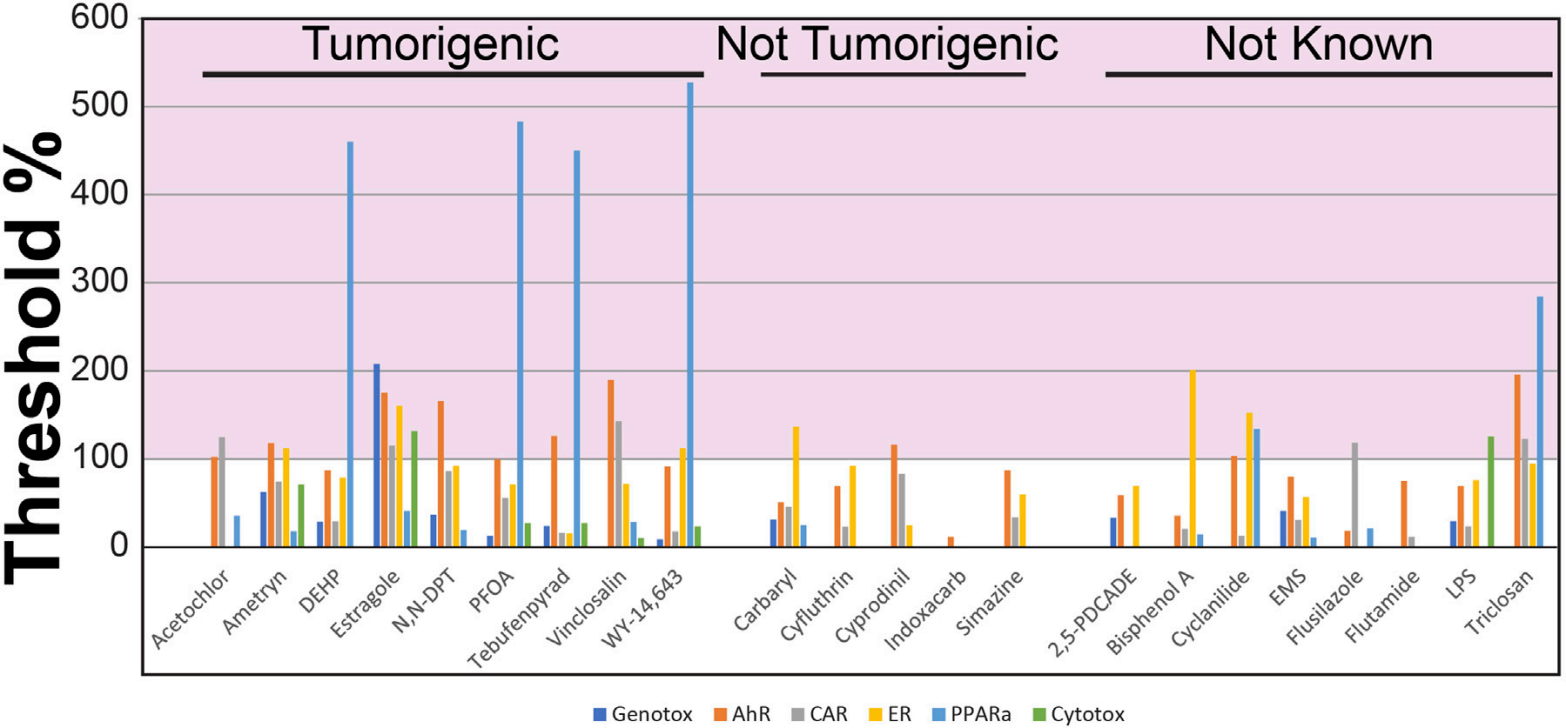
Identification of chemical-dose pairs that are tumorigenic. The 6 biomarkers were compared to the transcript profiles derived from the livers of rats exposed to the indicated chemicals (dose levels described in Table 1). The 6 -Log (*p*-value)s representing the correlation of each chemical to the 6 biomarkers was compared to the tumorigenic thresholds derived from the TG-GATES study. Values on the *y*-axis represent the (biomarker -Log (*p*-value)/the tumorigenic threshold) × 100. Any treatment that exceeds 100% for any of the biomarkers (pink shaded area) would be predicted to cause increases in liver tumors under chronic conditions.

To accomplish our first objective, we evaluated the transcript profiles derived from the livers of rats treated with 22 chemicals at one dose level for 4 days (Study A). These chemicals included pesticides, industrial chemicals and reference chemical activators of one or more MIEs. Each dose level was classified as tumorigenic, not tumorigenic or not known. There were 14 chemical-dose pairs that could be annotated for cancer outcome. Each profile was compared to the set of 6 biomarkers using the Running Fisher test. The level of activation of each biomarker was compared to the TAL derived from the TG-GATES study (TG-TAL) (Figure 2) or from the DrugMatrix study (DM-TAL) (Supplementary File S4). Figure 2 shows the TG-TALs relative to the tumorigenic levels for each of the biomarkers for the chemicals. There were 9 chemicals that were examined at tumorigenic dose levels (acetochlor, ametryn, di (2-ethylhexyl) phthalate (DEHP), estragole, N,N-dimethyl-p-toluidine, perfluorooctanoic acid (PFOA), tebufenpyrad, vinclozalin, WY-14,643 (WY)). DEHP and PFOA activated only one MIE (PPARα) at tumorigenic levels. The other chemicals activated a mixture of two or more MIEs but most commonly, AhR and CAR. Using the DM-TALs, the analysis was repeated and is shown in Supplementary File S4. The MIEs that were activated to tumorigenic levels were similar to the analysis with the TG-TALs. However, two chemicals were called false negatives as they were not correctly identified as tumorigenic (acetochlor, ametryn).

There were 5 chemicals examined at doses that did not induce liver tumors at the highest dose tested (carbaryl, cyfluthrin, cyprodinil, indoxacarb, simazine). Using the TG-TAL, cyprodinil was predicted to cause liver tumors through an AhR mechanism and carbaryl through an ER mechanism (false positives) (Figure 2). Using the DM-TAL, no chemicals were predicted to be tumorigenic (Supplementary File S4).

In addition to the tumorigenic and nontumorigenic chemicals, there were 8 chemicals (2,5-pyridinedicarboxylic acid, dipropyl ester (2,5-PDCADE), bisphenol A, cyclanilide, ethyl methanesulfonate (EMS), flusilazole, flutamide, lipopolysaccharide (LPS), triclosan) that could not be classified for tumorigenicity, either because the dose examined in the study was higher than the highest nontumorigenic dose or that the chemical had not been examined in a chronic study. For all but EMS, flutamide and 2,5-PDCADE, the chemicals were predicted to increase liver tumor incidence after 2 years using the TG-TALs (Figure 2), while only cyclanilide and triclosan would be predicted to cause liver tumors in chronic studies using the DM-TALs (Supplementary File S4).

We determined how accurate the NAM was at identifying chemical doses that were tumorigenic. The predictive accuracy using the TG-TALs was 80% (100% sensitivity; 60% specificity) (Table 4). The predictive accuracy using the DM-TALs was 89% (78% sensitivity; 100% specificity). The level of accuracy for this set of chemicals is within the range of accuracies demonstrated in previous studies.

**TABLE 4:** Predictive accuracies derived using the NAM.

Study	Unit of prediction	Tumorigenic activation level	Total number of biosets or chemicals examined	TP	TN	FP	FN	Sensitivity	Specificity	PPV	NPV	Balanced accuracy
Study A	Chemical-Dose	TG-GATES	14	9	3	2	0	1	0.6	0.82	1	0.8
Study A	Chemical-Dose	DrugMatrix	14	7	5	0	2	0.78	1	1	0.71	0.89
Study C	Chemical-Dose	TG-GATES	100	31	51	7	11	0.74	0.88	0.82	0.82	0.81
Study C	Chemical-Dose	DrugMatrix	100	22	56	2	20	0.52	0.97	0.92	0.74	0.75
Study C	Chemical	TG-GATES	16	11	3	2	0	1.00	0.60	0.85	1.00	0.80
Study C	Chemical	DrugMatrix	16	9	5	0	2	0.82	1.00	1.00	0.71	0.91

### 3.3 Relationships between biomarker TALs in affymetrix and RNA-Seq profiles

**FIGURE 3:**
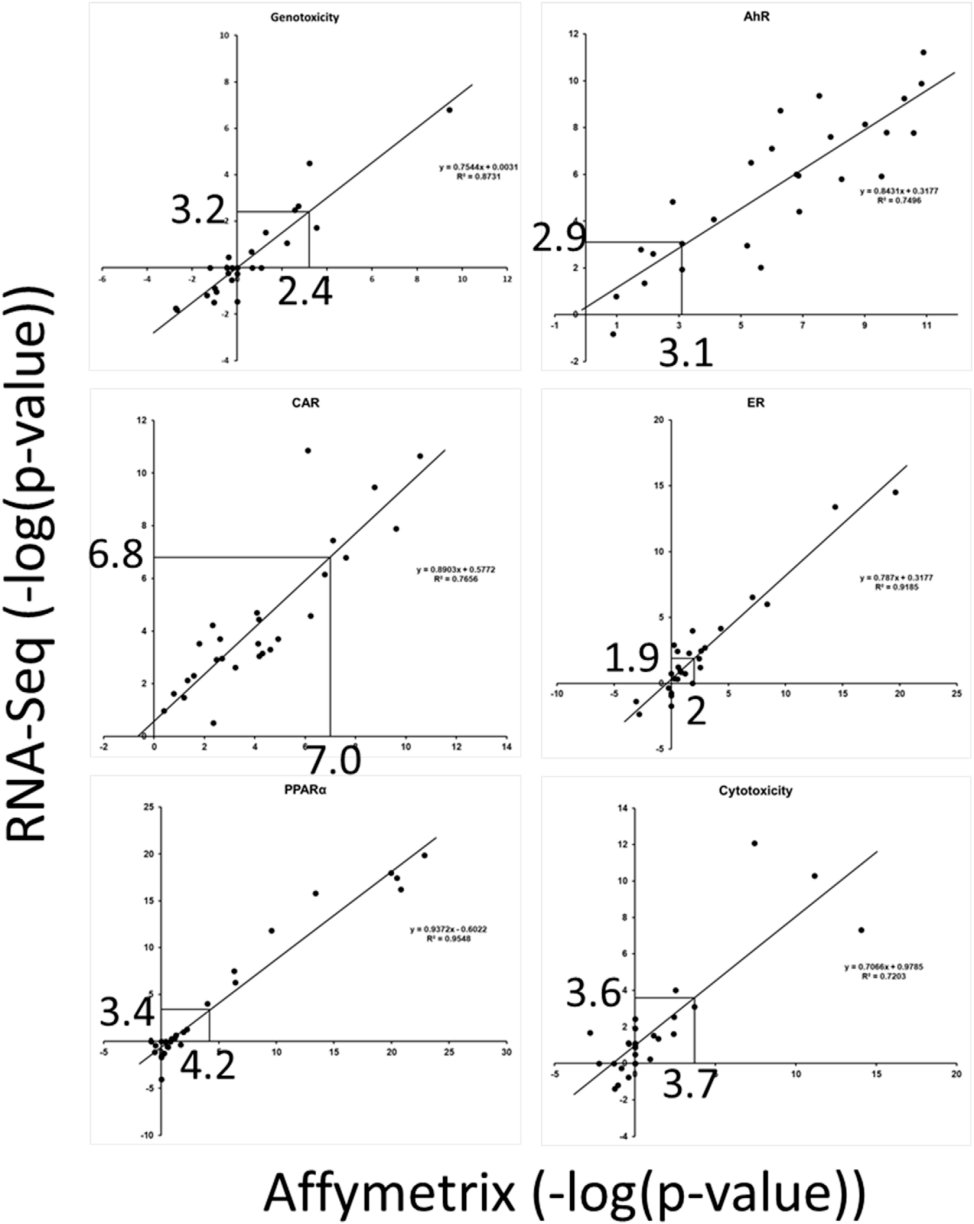
Relationships between biomarker activation levels derived using Affymetrix vs. RNA-Seq. Transcript profiles generated using either Affymetrix arrays or RNA-Seq were derived from the same livers of rats exposed to 27 chemicals. The pairs of profiles were compared to each biomarker. The TG-TALs are indicated on the *x*-axes and the derived TALs from the RNA-Seq analysis are shown on the *Y*-axes. The lines indicate linear trendlines. The figures show that the TALs derived from the Affymetrix data are similar to values derived from the RNA-Seq studies.

Our second objective was to determine if the biomarker TALs derived from microarray data could be applied to RNA-Seq data (Study B). A unique dataset was used to make comparisons between the two platforms. Male rats were exposed to 27 chemicals at one dose level each day for 3, 5, or 7 days, and the liver RNAs were evaluated using Affymetrix arrays and RNA-Seq. The two transcript profiles from each chemical-dose pair were compared to the set of 6 biomarkers. Figure 3 shows the biomarker activation levels (using -Log (*p*-value)s of the Running Fisher test as metrics) across all of the chemicals for individual biomarkers. The figures show that for the most part, there is a linear relationship between the activation levels determined by Affymetrix arrays and by RNA-Seq, especially within the range of the biomarker TALs (∼2–7). Using the TG-TALs (Figure 3), we found that the levels derived from extrapolation to the RNA-Seq data were similar. For all but genotoxicity, the TALs from the RNA-Seq data were somewhat smaller compared to the TALs derived from the Affymetrix data. Using the DM-TALs (Supplementary Figure S4), we found that the levels derived from extrapolation to the RNA-Seq data were also similar. The findings indicate that the TALs could potentially be used to make predictions using RNA-Seq data.

### 3.4 Identification of chemical-dose pairs that are tumorigenic using TempO-Seq data

**FIGURE 4:**
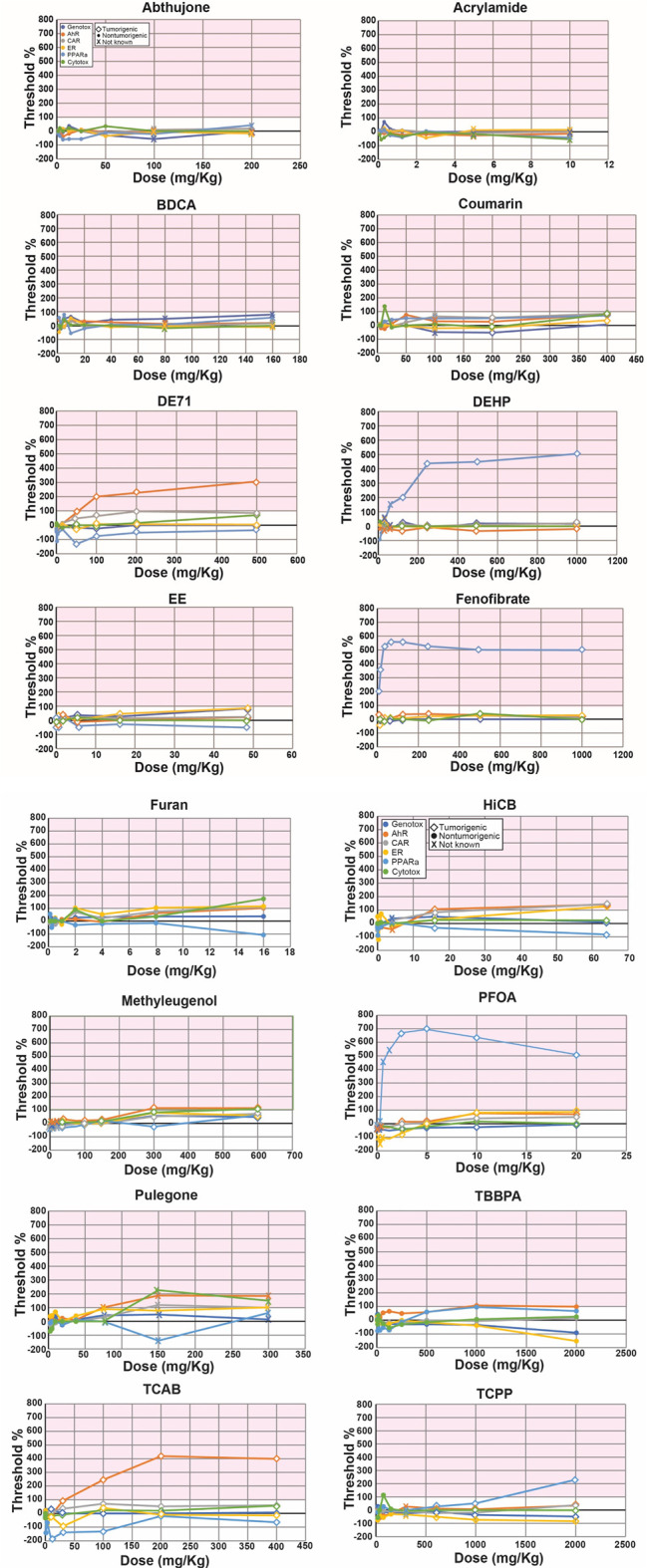
Biomarker activation levels identify chemical-dose pairs that are tumorigenic in chronic studies. Rats exposed to 16 chemicals at up to 10 dose levels were evaluated for gene expression changes using targeted RNA-Seq (TempO-Seq). Each derived gene list was compared to the 6 biomarkers using the Running Fisher test. Dose-dependent changes in the -Log (*p*-value)s of each biomarker relative to the derived TG-TALs are shown. A similar analysis using the DrugMatrix TALs is found in Supplementary File S4. The TAL for each biomarker was set at 100%. The different color lines track the changes in the TALs for each of the molecular initiating events. Each dose is indicated as a diamond (tumorigenic), a filled circle (not tumorigenic) or x (tumorigenicity at this dose is not known). Abbreviations: AhR, aryl hydrocarbon receptor; CAR, constitutive activated receptor; ER, estrogen receptor; PPARα, peroxisome proliferator-activated receptor α.

There have been no studies applying the NAM to full-genome TempO-Seq-derived transcript profiles to make predictions. We utilized a dataset from the livers of rats treated with 16 chemicals for 5 days at up to 10 dose levels for a total of 132 chemical-dose comparisons (Study C). There were 100 comparisons that could be annotated for potential to induce tumors. Figure 4 shows the biomarker activation levels relative to the TG-TALs for each chemical. The figures derived from the analysis using the DM-TALs are shown in Supplementary File S4.

Predictive accuracies were determined two ways. In the first method, accuracy was based on the 100 chemical-dose pairs that could be annotated for chronic outcomes. Table 4 shows that the balanced accuracies using the TG-TALs or DM-TALs was 81% or 74%, respectively. Using the TG-TAL, there were 11 false negatives for 4 chemicals (furan, TCAB, EE, methyleugenol) and 7 false positives for 6 chemicals (coumarin, TCPP, BDCA, BDCA, TBBPA, coumarin, fenofibrate). A number of the false positives were at doses lower than those that were tumorigenic including for coumarin, DE71, fenofibrate, and TCPP, indicating the TG-TALs are sensitive to gene changes that precede overt tumor induction. Using the DM-TALs, there were 20 false negatives for 8 chemicals (coumarin, DE71, EE, furan, HCB, methyleugenol, TCAB, TCPP) and 2 false positives for 2 chemicals (coumarin, fenofibrate). The relatively high level of false negatives compared to the TG-TALs may be due to the higher -Log (*p*-value)s for the DM-TALs for all 6 biomarkers.

In a screening study to identify hazards, all doses would be considered, not just individual chemical-dose pairs. When the accuracy was determined based on evaluation of all dose levels for each chemical, the balanced accuracies were 80% and 91% for the TG- and DM-TALs, respectively. There were 2 false positives (BDCA, TBBPA) using the TG-TALs and no false negatives. For BDCA the TALs were not dose-dependent; the activation levels were achieved at 10 mg/kg for ER and at 5 mg/kg for PPARα. This is in contrast to the true positive chemicals in which there was usually more than one dose level that was positive for one of the MIEs and occurred at the higher dose levels. There were 2 false negatives (EE, methyleugenol) using the DM-TALs and no false positives. Thus, the NAM can be accurately applied to TempO-Seq data.

## 4 Discussion

New approach methodologies (NAMs) have the potential to radically transform carcinogenicity testing. Integrated sets of *in vitro* assays could be used in IATA-type approaches. However, their ability to accurately predict cancer has not been fully tested. Short-term exposures in test species coupled with NAMs have the potential to greatly reduce the number of animals and could act as a bridge between the current requirements for chronic exposure testing and future *in vitro* testing strategies. Here, we describe a novel NAM that can be used with transcript profiling measurements to identify in short-term exposures, chemicals and their doses that would cause tumors in the livers of rats (Figure 1). Capitalizing on three studies conducted in rats in which liver gene expression was evaluated after 3–7 days exposures, we demonstrated that 1) using Affymetrix data, the NAM could identify individual chemical-dose pairs that were tumorigenic (80% or 89% accuracy); 2) when comparing the transcript profiles generated from the same liver samples by Affymetrix and RNA-Seq, there were no notable differences in the responses in the -Log (*p*-value) range of biomarker TALs, indicating the TALs derived from microarray data could be applied to RNA-Seq data, and supporting this observation; 3) using TempO-Seq-generated transcript profiles, the NAM was able to identify chemicals and their dose levels that would be tumorigenic with 75%-91% accuracy. In summary, the NAM can be used for prediction of liver tumor induction under different rat exposure scenarios and using different platforms to interrogate RNA expression.

Due to the diversity and complexity of the biological processes underlying tumor formation, the ability to predict human tumor induction using sets of nonanimal-based NAMs within an IATA framework will be challenging. While rodent tumor formation does not always mimic that in humans, regulatory mandates require rodent carcinogenicity testing, which the current study is meant to support and optimize. While approaches using large sets of *in vitro* assays coupled with *in vitro* to *in vivo* extrapolation to set exposure limits appear to be promising (Paul Friedman et al., 2020), most new chemicals will not be evaluated using even a subset of the assays. Short-term tests in animals that link molecular and cellular changes to subsequent toxicity may provide a way to reduce animal testing, especially if approaches for harmonization of animal tests could be agreed upon. Use of HTTr gives a better understanding of underlying toxicity by indicating the actual toxicological mechanisms, which can be used to infer eventual toxicity and carcinogenesis; thus, this allows for the use of shorter exposures on fewer animals by negating the need to wait for the possible development of cancers over a rodent’s lifetime. This approach could be incorporated into new standards to make future animal use more reliable and relevant, whilst reducing animal usage and suffering overall, and falling in step with the 3Rs of toxicology. With this in mind, an approach that has been receiving much attention recently is the *in vivo* application of transcriptomics for establishing a “bioactivity” point of departure (PoD). This approach is based on the hypothesis that any toxicity (including carcinogenicity) is not likely to occur in the absence of changes in gene expression in one or several sentinel tissues (Thomas et al., 2013) (https://www.epa.gov/etap). Promising studies examining adult and fetal tissues (e.g., Johnson et al., 2022) after short-term exposures have shown that the derived PoD could be used to protect human populations from adverse effects. The EPA has proposed to use this approach to determine PoD based on transcriptomics for data-poor chemicals (https://www.epa.gov/etap). Implementing this strategy to large sets of chemicals will be challenging due to the costs of the studies, identification of appropriate exposure conditions, the choice of tissues to examine, and the computational methods for deriving the PoD. Despite these challenges, the approach has the potential to greatly reduce the animal requirements for not only the 2-year bioassay, but other animal tests required by regulatory agencies. Until the toxicity testing community has greater confidence in this approach, NAMs with known predictive accuracies for important endpoints will likely assist in making regulatory decisions.

The NAM approach described and tested here was built using the network of liver cancer AOPs as a starting point that can be found in the AOPWiki (https://aopwiki.org/). The importance of using the AOP framework for building and testing NAMs is highlighted by work in which knowledge related to carcinogenicity assessment has been reorganized into AOP networks resulting in the development of the Kaptis model (https://www.lhasalimited.org/products/kaptis.htm) which like the AOPWiki has the potential to facilitate interpretation of the weight of evidence of available information related to carcinogenicity assessment and future integration of existing and emerging *in vitro* and *in vivo* assays used for prediction (Felter et al., 2021). While each of the liver cancer AOPs examined in our study contain key events downstream of the MIEs, many of these KEs cannot be measured using transcript profiling. Thus, we originally focused on methods to predict each of the MIEs of the major liver cancer AOPs using transcript profiling, a now routine method for identifying chemical hazards. The 6 biomarkers were constructed using profiles derived from the livers of rats exposed to reference chemical activators of each of the MIEs. In our past studies, the individual gene expression biomarkers had balanced accuracies of 92%–98% (Rooney J. P. et al., 2018; Hill et al., 2020). We found in these studies that most chemicals have mixed MOAs in that they activate 2 or more MIEs under conditions that would cause cancer. This finding highlights the need for measurement of all MIEs when considering whether exposure to a chemical would be relevant to humans.

Our approach to determining the activation of MIEs is similar to that described by another group. Using a multivariate regression approach applied to liver RNA-Seq data derived from rats exposed to a diverse reference chemical set enabled the identification and refinement of gene sets (biomarkers) predictive of agonists for 5 different canonical xenobiotic receptors (AhR, CAR, Pregnane X Receptor [PXR], PPARα, ER), 3 mediators of reactive metabolite stress responses (NRF2, NRF1, p53), and activation of the innate immune response (Podtelezhnikov et al., 2020). Additionally, a composite transcriptional biomarker of tissue injury and regenerative repair response was described by the same group and could be applied across 8 different tissues (Glaab et al., 2021). These 10 biomarkers along with thresholds for AhR activation (Qin et al., 2019) are used by the group for routine monitoring in initial rat tolerability studies just prior to entering drug development to identify drug candidate potential for activating these MIEs to trigger liver and other organ toxicities with strong (>90%) sensitivity and/or specificity (Monroe et al., 2020; Glaab et al., 2021).

These AOP-based approaches to predicting toxicity and cancer are different from the key characteristics of carcinogens (KCC) approach (Smith et al., 2016; Guyton et al., 2018) originally inspired by the idea of the Hallmarks of Cancer (Hanahan and Weinberg, 2000; Hanahan and Weinberg, 2011) and identified and developed to organize new lines of evidence for assessing carcinogenicity. In the first study using the KCCs, Smith et al. (2016) analyzed the biological effects of chemicals classified as known human carcinogens and defined 10 KCCs. Tice et al. (Tice et al., 2021) reviewed the KCCs as a method to develop an IATA of carcinogenic potential using NAMs. However, their conclusion echoed by others (Goodman et al., 2018) was that the KCCs lack the necessary specificity for carcinogenicity prediction as KCCs are also involved in disease processes that are not related to cancer. Furthermore, no scheme has yet been proposed in which to relate the number of KCCs that are “positive” and carcinogenicity potential, the identification of assays to determine if the chemical exhibits that KCC, and how the KCCs could be used in a quantitative manner. There is general agreement that KCCs could play a role in assembling lines of evidence in assessing carcinogenic potential that would complement other relevant information.

While our MIE biomarkers had demonstrated utility in identifying chemical MOAs for liver tumorigens, it was not possible using the biomarkers alone to identify the doses of a chemical that would cause cancer. Thus, in later studies we capitalized on a central premise of the AOP concept which is that while MIEs/KEs are required at a qualitative level, they must be activated to a sufficient level and duration to cause an adverse outcome (Conolly et al., 2017). Computationally-derived quantitative effect levels, or “molecular tipping points” can be used as tools for adversity determinations using shorter-term data (Julien et al., 2009; Knudsen et al., 2015). Using biomarker TALs that were derived a number of ways, we found that across 163 chemicals examined at multiple time points, the NAM had predictive accuracies of 96%–97% (Corton J. C. et al., 2020; Lewis et al., 2020). We also found that data requirements for prediction could be reduced to measuring 12 individual genes (2 from each biomarker) (Corton J. et al., 2020), or measuring combinations of liver weight to body weight and clinical chemistry markers (Corton J. C. et al., 2020); these approaches were predictive of liver tumors at up to 97% balanced accuracy. These predictions were based for the most part on legacy microarray and associated data from TG-GATES and DrugMatrix datasets. Remarkably from the current study, we showed that the predictive accuracies using full-genome targeted RNA-Seq (TempO-Seq) transcript profile data was as high as 91%, in the same range as our original studies. Thus, our NAM can be used under a wide number of short-term exposure scenarios (4–29 days) using transcript profiling platforms that are more commonly in use today.

The 6 biomarkers and their TALs discussed here could be applied in a number of ways for toxicological testing of industrial chemicals. After preliminary short-term exposure studies followed by gene expression analysis, the TALs could be used to help bracket the range of doses between the BMD and the calculated dose that would be expected to induce liver tumors. Knowledge of the TALs could be used to allow informed decisions to be made of doses to use in chronic studies to avoid tumor induction. In testing for pharmaceutical candidates, the TALs could be used to support reduced carcinogenicity testing under the International Council for Harmonization of Technical Requirements for Pharmaceuticals for Human Use (ICH) S1 guidance modification initiatives. Modifications to ICH S1 Carcinogenicity Testing Guidance (ICH, 2015) proposes a more flexible approach to pharmaceutical carcinogenicity testing. This allows for adequate assessment of carcinogenic risk without the need for always conducting a 2-year rat carcinogenicity study. This modification in the guidance may enable drug sponsors to gain 2-year rat carcinogenicity study waivers through a Carcinogenicity Assessment Document (CAD)-based justification process. Our study represents an example of how gene expression thresholds could be leveraged as “new biomarkers” data (ICH, 2015) to strengthen CAD-based predictions. If, for example, after a 6-month study there are histopathological indicators of liver cancer signals, a short-term toxicogenomic study coupled with our biomarker TAL approach would provide information about the underlying AOP and doses that would lead to liver tumors and possibly contributing to the conclusion that a 2-year bioassay is not needed.

Given the convergence of approaches to build and utilize gene expression biomarkers by multiple groups, the HESI Emerging Systems Toxicology for the Assessment of Risk (eSTAR) committee has an ongoing multi-institution effort to identify predictive gene sets. The committee will employ a number of computational approaches to liver transcript profiles of chemicals annotated for liver cancer MIE modulation and cancer outcomes (Corton et al., 2022a). The approach will include data from wild-type *versus* factor-null rats where gene dependence on ligand-activated transcription factors can be confirmed, complemented with a large body of published or to-be-generated data including ChIP-Seq data to further support specific compound MIEs. The hope is that scientific consensus between investigators will result in a validated set of biomarkers and computational techniques that will be accepted by various regulatory agencies for widespread use for internal decision making as well as for regulatory applications.

In summary, the NAM described and tested here to be used to replace carcinogenicity testing exhibits characteristics desired in a method used for prediction. These include accurate prediction of whether the MIE is modulated and most importantly, whether the dose of the chemical would be tumorigenic in chronic studies. The NAM could be used for screening chemicals in short-term exposures to identify potential liabilities or after a chronic study before the appearance of tumors when the liver is found to be a tissue with histopathology findings of concern. The continued use of *in vivo* tests using new animal models or modifications of existing guideline animal studies of shorter duration is increasingly recognized as a necessity for bridging gaps en route to establishing new animal-free regulatory frameworks that are the goal of regulatory Agencies worldwide.

## Data Availability

The datasets presented in this study can be found in online repositories. The names of the repository/repositories and accession number(s) can be found in the article/Supplementary Material.

## Supplementary material

SUPPLEMENTARY FILE S1 Carcinogenicity information for the chemicals used in the rat 4-day study.

SUPPLEMENTARY FILE S2 Carcinogenicity information for the chemicals used in the rat 5-day study.

SUPPLEMENTARY FILE S3 Tumorigenic activation levels.

SUPPLEMENTARY FILE S4
Supplementary Figures S1–S3.
