# Supplementary material for: A new approach methodology to identify tumorigenic chemicals using short-term exposures and transcript profiling
Source: Front Toxicol. 2024 Oct 17;6:1422325. doi: 10.3389/ftox.2024.1422325 (PMC11526388; doi:10.3389/ftox.2024.1422325)
Supplement: Supplementary file 2 [file Table1.docx]

**Supplementary information:**

**A new approach methodology to identify tumorigenic chemicals**

**using short-term exposures and transcript profiling**

Victoria Ledbetter^1,2^, Scott Auerbach^3^, Logan Everett^1^, Beena Vallanat^1^, Anna Lowit^4^, Gregory Akerman^4^, William Gwinn^3^, Leah C. Wehmas^1^, Michael F. Hughes^1^, Michael Devito^1^, J. Christopher Corton^1,5^

^1^Center for Computational Toxicology and Exposure, US Environmental Protection Agency, Research Triangle Park, North Carolina 27711, USA.

^2^Oak Ridge Associated Universities (ORAU), Oak Ridge, Tennessee 37830, USA.

^3^National Institute of Environmental Health Sciences (NIEHS), National Toxicology Program (NTP), Research Triangle Park, North Carolina 27709, USA.

^4^U.S. Environmental Protection Agency, Office of Pesticide Programs, MC7507P, 1200 Pennsylvania Avenue NW, Washington, DC, 20460, USA.


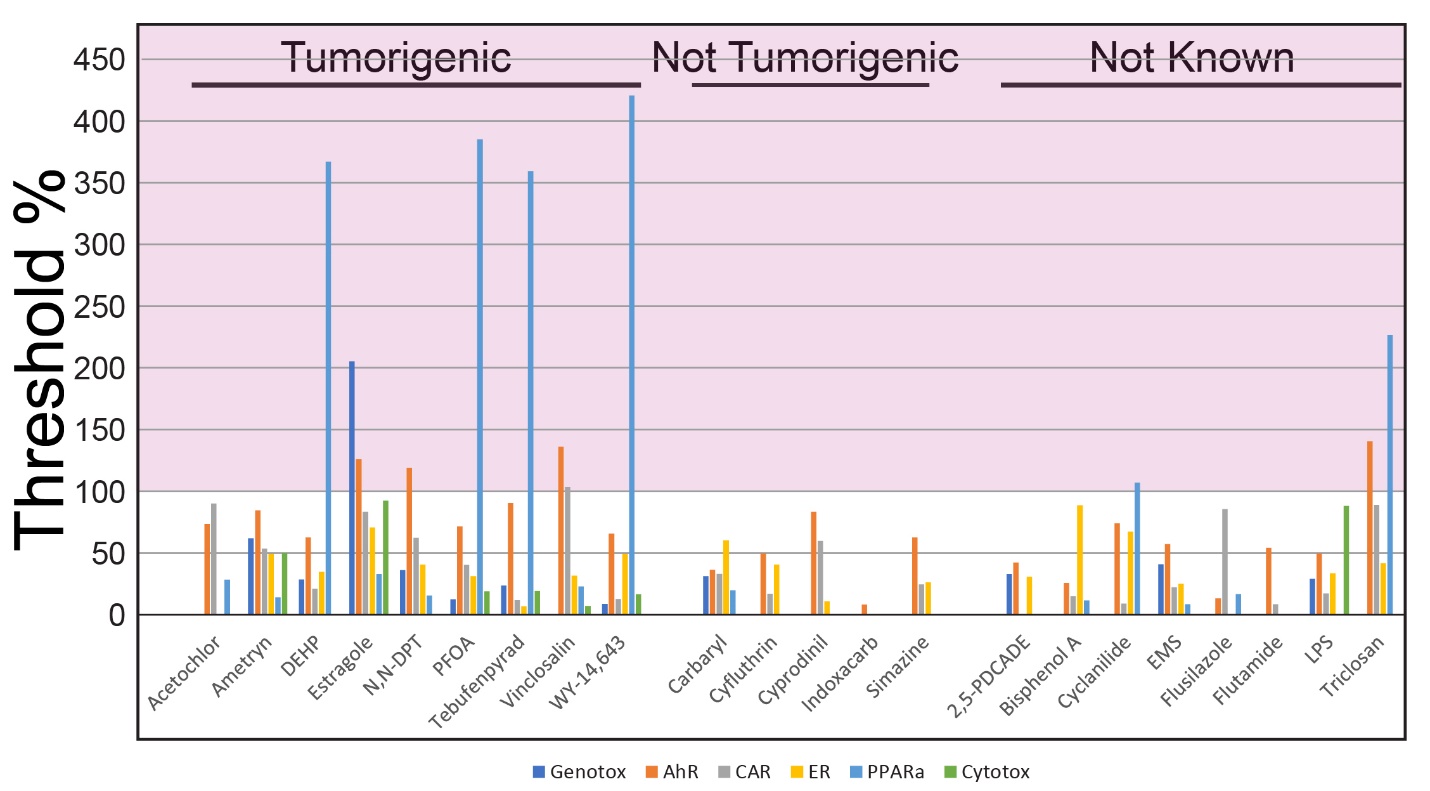


**Fig. S1. Identification of chemical-dose pairs that are tumorigenic.**

The 6 biomarkers were compared to the transcript profiles derived from the livers of rats exposed to the indicated chemicals (dose levels described in **Table 1**). The 6 -Log(p-value)s representing the correlation of each chemical to the 6 biomarkers was compared to the tumorigenic thresholds derived from the DrugMatrix study. Values on the y axis represent the (biomarker -Log(p-value)/the tumorigenic threshold) x 100. Any treatment that exceeds 100% for any of the biomarkers (pink shaded area) would be predicted to cause increases in liver tumors under chronic conditions.


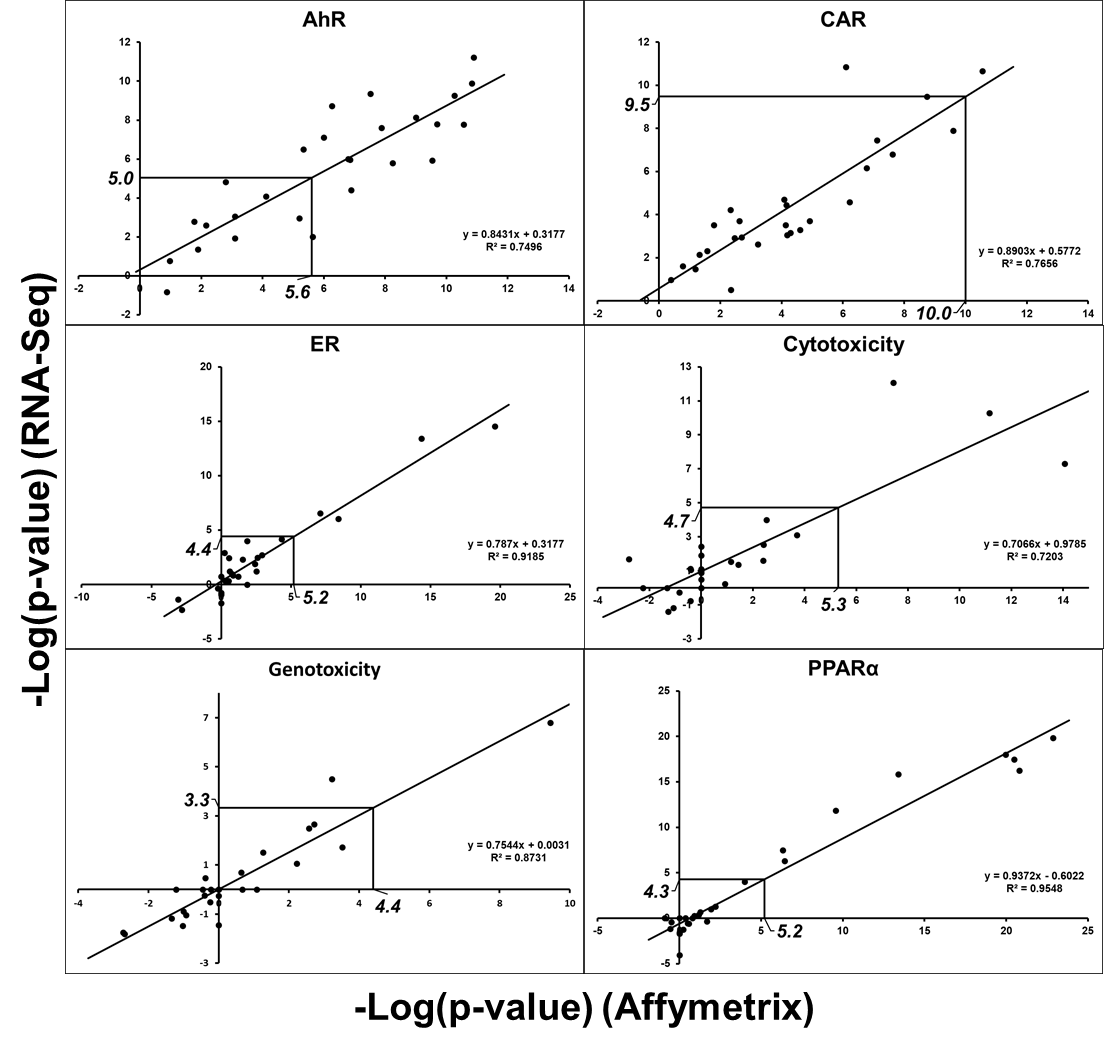


**Fig. S2. Relationships between biomarker activation levels derived using Affymetrix vs. RNA-Seq.**

Transcript profiles generated using either Affymetrix arrays or RNA-Seq were derived from the same livers of rats exposed to 27 chemicals. The pairs of profiles were compared to each biomarker. The DM-TALs are indicated on the x-axes and the derived TALs from the RNA-Seq analysis are shown on the Y-axes. The lines indicate linear trendlines.


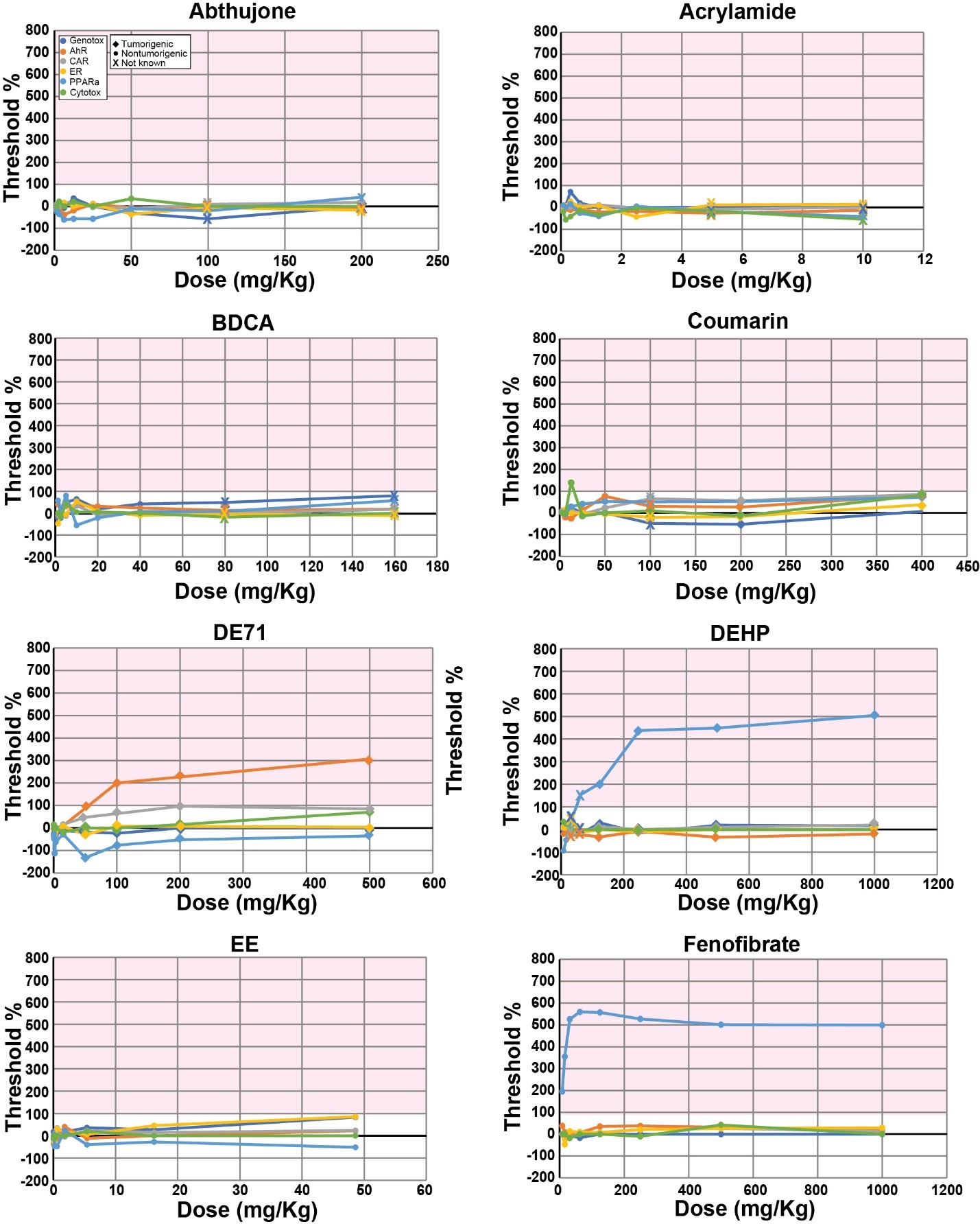


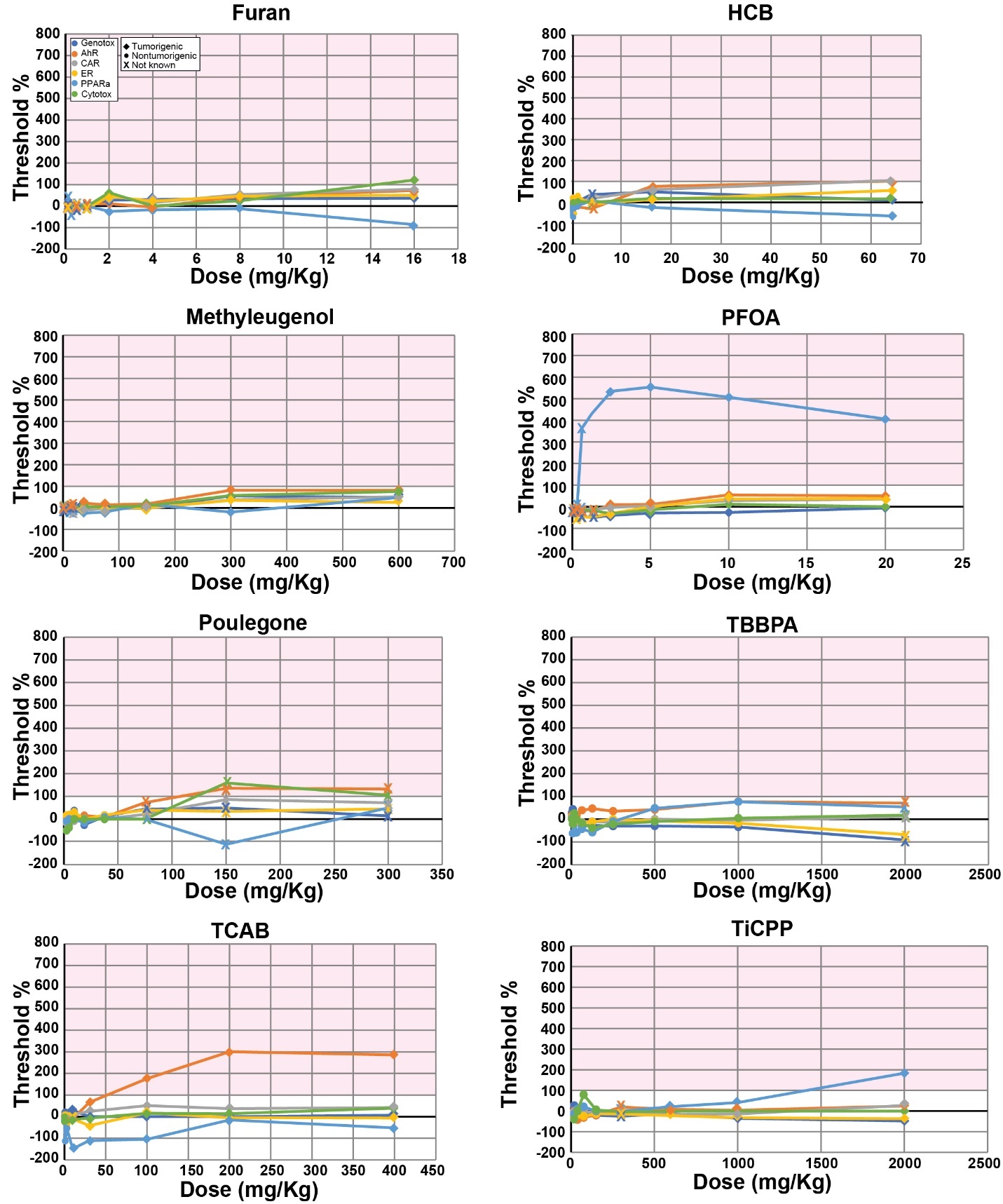


**Fig. S3. Biomarker activation levels identify chemical-dose pairs that are tumorigenic in chronic studies.**

Rats exposed to 16 chemicals at up to 10 dose levels were evaluated for gene expression changes using targeted RNA-Seq (TempO-Seq). Each derived gene list was compared to the 6 biomarkers using the Running Fisher test. Dose-dependent changes in the -Log(p-value)s of each biomarker relative to the derived DM-TALs are shown. The TAL for each biomarker was set at 100%. The different color lines track the changes in the TALs for each of the molecular initiating events. Each dose is indicated as a diamond (tumorigenic), a filled circle (not tumorigenic) or x (tumorigenicity at this dose is not known). Abbreviations: AhR, aryl hydrocarbon receptor; CAR, constitutive activated receptor; ER, estrogen receptor; PPARα, peroxisome proliferator-activated receptor α.
